# Baicalin administration could rescue high glucose-induced craniofacial skeleton malformation by regulating neural crest development

**DOI:** 10.3389/fphar.2024.1295356

**Published:** 2024-03-07

**Authors:** Jia-Qi Lu, Zhi-Yan Luo, Chengyang Sun, Si-Miao Wang, Dixiang Sun, Ruo-Jing Huang, Xuesong Yang, Yong Ding, Guang Wang

**Affiliations:** ^1^ The First Affiliated Hospital of Jinan University, Jinan University, Guangzhou, China; ^2^ Division of Histology and Embryology, International Joint Laboratory for Embryonic Development and Prenatal Medicine, School of Medicine, Jinan University, Guangzhou, China; ^3^ Department of Pathology, Mengyin County Hospital of Traditional Chinese Medicine, Linyi, China; ^4^ Key Laboratory for Regenerative Medicine of the Ministry of Education of China, Jinan University, Guangzhou, China

**Keywords:** baicalin, diabetes mellitus, hyperglycemia in pregnancy, NCC delamination and migration, craniofacial skeleton

## Abstract

Hyperglycemia in pregnancy can increase the risk of congenital disorders, but little is known about craniofacial skeleton malformation and its corresponding medication. Our study first used meta-analysis to review the previous findings. Second, baicalin, an antioxidant, was chosen to counteract high glucose-induced craniofacial skeleton malformation. Its effectiveness was then tested by exposing chicken embryos to a combination of high glucose (HG, 50 mM) and 6 μM baicalin. Third, whole-mount immunofluorescence staining and *in situ* hybridization revealed that baicalin administration could reverse HG-inhibited neural crest cells (NCC) delamination and migration through upregulating the expression of Pax7 and Foxd3, and mitigate the disordered epithelial-mesenchymal transition (EMT) process by regulating corresponding adhesion molecules and transcription factors (i.e., E-cadherin, N-cadherin, Cadherin 6B, Slug and Msx1). Finally, through bioinformatic analysis and cellular thermal shift assay, we identified the AKR1B1 gene as a potential target. In summary, these findings suggest that baicalin could be used as a therapeutic agent for high glucose-induced craniofacial skeleton malformation.

## Introduction

The generation of neural crest cells (NCCs) occurs when primary neurulation occurs, i.e., the process of dorsal fusion of the neural plate borders (Osório et al., 2004). Neurulation is defined as the morphogenetic process in which the neural plate transforms into a neural tube, and then the neural progenitor cells in the neural tube differentiate into the cellular components of the brain and the spinal cord (Colas and Schoenwolf, 2001). Failure of primary neurulation, i.e., defective formation of neural tube, will lead to malformations known as neural tube defects (NTDs), the second most prevalent congenital anomaly (Copp et al., 2003; Mitchell, 2005). It was reported that NTDs were closely associated with the abnormal development of NCCs (Stevenson et al., 2004; Wang et al., 2016). During early vertebrate development, NCCs are derived from the dorsal-most aspect of the neural tube (Achilleos and Trainor, 2012). Thereafter, primitive NCCs undergo a succession of events in chronological order, including NCC induction, delamination, epithelial-mesenchymal transition (EMT), migration and differentiation, and eventually contribute a variety of NCC-derived cells (Zhang et al., 2017). Obviously, NCCs are endowed with the remarkable ability of migration and differentiation, thereby making up various cells and tissues of the neural crest (Takahashi et al., 2013; Simões-Costa and Bronner, 2015). The neural crest derivatives comprise the whole craniofacial skeleton, cerebral ganglions, enteric nervous system, and Schwann cells, while cranial NCCs contribute cranial bones, cartilage, smooth muscle, dentin, melanocytes, corneal endothelial cells, and neurons and glial cells in the peripheral nervous system (Cordero et al., 2011; Ishii et al., 2012). Regarding the regulation of NCC development, the interaction of transcription factors and cell signaling molecules has been suggested to involve the aforementioned morphogenetic formation, such as bone morphogenetic proteins (BMPs), Snai2 (Slug), Wnts, and fibroblast growth factors (FGFs), etc. (Barembaum and Bronner-Fraser, 2005). Given the close correlation between NCC development and NTDs (Takahashi et al., 2013; Reid et al., 2015), we investigated the treatment strategy for alleviating NCC malformation resulting from hyperglycemia in pregnancy (HIP) using chicken embryos, an early developmental vertebrate model.

HIP can cause very serious pregnancy-specific health problems (Negrato and Gomes, 2013). HIP is generally classified as either diabetes mellitus in pregnancy (DIP) or gestational diabetes mellitus (GDM), which is deemed damaged glucose tolerance during pregnancy. In other words, HIP could be regarded as a consequence of either preexisting diabetes or GDM (López Stewart, 2014; Hod et al., 2015). Approximately 80% of diabetic pregnancies are vested in GDM (Virjee et al., 2001). Noticeably, GDM is usually not diagnosed in the clinic until the 24th to 28th week of gestation, but then it is too late to implement treatment since the most important period for embryonic/fetal development is the early stage of embryo development. Thus, maternal HG could have already impacted the early development of the fetus. It was reported that maternal hyperglycemia greatly increased the risk of congenital abnormalities in the cardiovascular and nervous systems. For example, hyperglycemia could lead to NTDs, including exencephaly, anencephaly and rachischisis (Dheen et al., 2009; Sukanya et al., 2012). Up to 17% of neonates of diabetic mothers suffer from congenital heart diseases (e.g., atrioventricular septal defects (AVSDs) and tetralogy of Fallot) (Corrigan et al., 2009). Obviously, understanding and treating diabetes during pregnancy would help prevent or lower maternal and fetal complications (Negrato and Gomes, 2013).

As a flavone glycoside (i.e., glucuronide of baicalein), baicalin is produced from the combination of glucuronic acid and baicalein. In nature, baicalein exists in the roots of Scutellaria lateriflora and Cutellaria baicalensis, which have been used as herbal medicine to prevent miscarriage by improving the developmental competence of embryos (Abel, 1984; Qi et al., 2016; Preventive et al., 2023). Furthermore, in most Asian countries, baicalin is also largely used as an herbal supplement because of its broad scope of health benefits, such as anti-neuroinflammation (Wang et al., 2016), anticancer (Wang et al., 2015), anti-anxiety (Wang et al., 2008), and increased lung capability (Baek et al., 2014) as well as fertility (Zhang et al., 2015). Regarding its biological mechanism, baicalin is believed to perform effectively by suppressing oxidative stress (Yin et al., 2011). Our previous study showed that baicalin administration could attenuate HG-induced malformations of the cardiovascular and nervous systems during embryonic/fetal development (Wang et al., 2018a; Wang et al., 2020). However, whether baicalin can prevent or rescue the malformation of the neural crest-derived craniofacial skeleton under HG conditions remains elusive. Thus, this study focused on the beneficial effects of baicalin and its corresponding mechanism on challenging HG-induced damage to neural crest development using early chick embryos as an experimental model, which has been previously proven effective (Wang et al., 2015; Wang et al., 2018a).

## Materials and methods

### Meta-analysis

**FIGURE 1:**
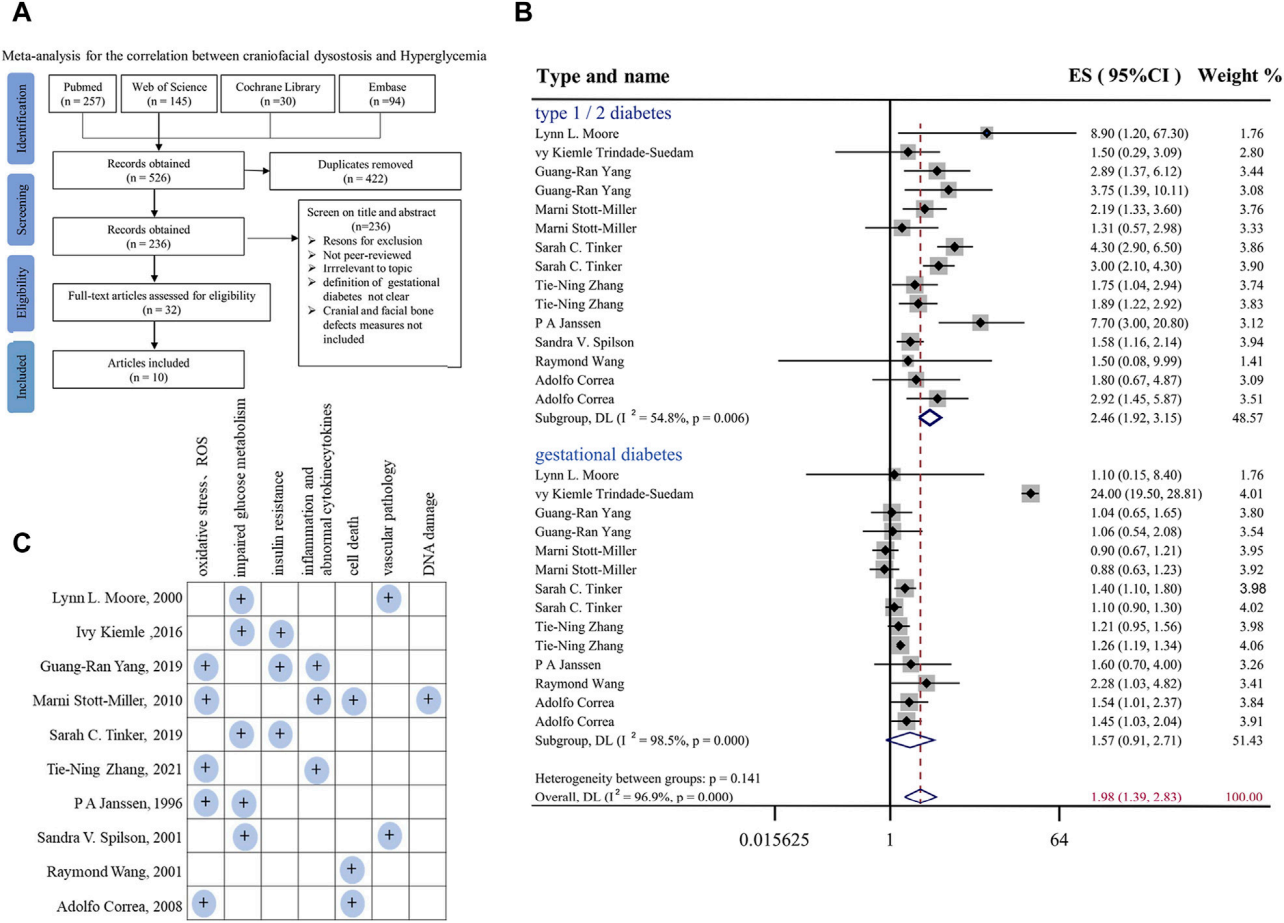
Meta-analysis for the correlation between hyperglycemia in pregnancy and craniofacial anomalies **(A)** Flow diagram of systematic literature search on hyperglycemia and craniofacial skeleton malformations. **(B)** Forest plot of effect sizes of studies examining the association of hyperglycemia in pregnancy and craniofacial anomalies included in the meta-analysis. Lowercase letters following the study year represent study arms within the same research. Effect size estimates (ES) are depicted by filled circles, with horizontal whiskers corresponding to 95% CIs. The length of the horizontal lines represents the 95% CIs for the effect size of hyperglycemia in pregnancy on craniofacial anomalies from each study. The weight of each study assigned to the meta-analysis expressed as a percentage is listed in the last column. The vertical solid line represents a mean difference of zero or no effect. I^2^ is a measure of variation beyond chance among studies used in the meta-analysis. The hollow diamond indicates the overall mean effect size. **(C)** Summary and classification of potential mechanisms of craniofacial abnormalities caused by hyperglycemia during pregnancy mentioned in 10 included studies. Abbreviations: ES, effect size; CI, confidence interval.

The meta-analysis was carried out using Stata version 13.1 (Stata Corp, College Station, TX, United States). The literature search was performed by querying PubMed, Embase, Cochrane Library and Web of Science for studies up to 1 January 2022, and the scientific question focused on the association between craniofacial anomalies and maternal diabetes. The flowchart of the search process is shown in Figure 1A. The inclusion criteria were as follows (Osório et al., 2004): articles reporting information on the association between gestational diabetes and craniofacial anomalies (Colas and Schoenwolf, 2001); articles in English or another European language; and (Copp et al., 2003) study design: cross-sectional studies, case‒control studies, epidemiologic studies, population-based studies, observational studies, clinical trials or cohort studies. The articles were excluded if they (Osório et al., 2004) did not investigate the aims of the study (Colas and Schoenwolf, 2001); did not provide an explicit definition of gestational diabetes (Copp et al., 2003); lacked cranial and facial bone defect definitions; or (Mitchell, 2005) did not report original data (e.g., editorial, review or congress abstract).

A summary of the studies included in the meta-analysis is reported in Supplementary Table S1. For the risk of bias assessment, the quality of the studies was evaluated independently by the two assessors via the qualitative evaluation of observational studies, i.e., Newcastle Ottawa Scale (NOS). The Newcastle Ottawa Scale is shown in Supplementary Tables S2, S3. Any disagreement in quality assessment was resolved through consensus. Studies scoring >7 were considered at low risk of bias, scores between 5 and 7 indicated a moderate risk of bias, and scores below 5 indicated a high risk of bias. Heterogeneity among studies was assessed by using the Q and I^2^ statistics. For the Q statistic, statistical significance was set at *p* < 0.1. Publication bias was evaluated using funnel plots and Egger’s regression test, and the results are shown in Supplementary Figures S1A, B. *p* < 0.1 was considered to indicate statistical significance.

### Avian embryos and treatment

**FIGURE 2:**
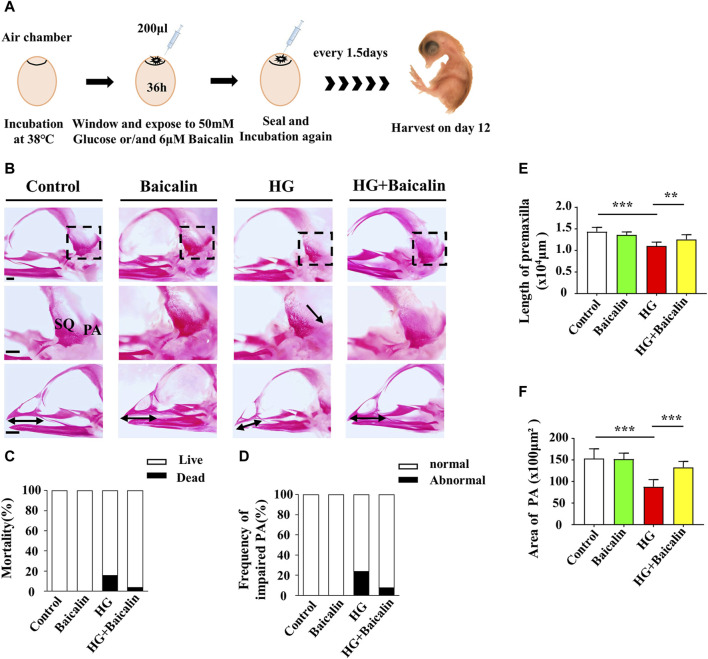
Assessing the development of the skulls of chicken embryos exposed to HG and/or baicalin **(A)** A schematic illustration showing that chicken embryos (preincubated for 1.5 days) were injected with an equal volume of mannitol (control, 50 mM HG, or/and 6 μM baicalin) every 3 days and then incubated at day 12. **(B)** Representative skull images of alizarin red-stained chicken embryos from the control, Baicalin, HG, and HG + Baicalin groups. **(C)** Bar charts showing the comparisons of the mortality of 12-day chick embryos among the control, Baicalin, HG, HG + Baicalin groups. **(D–F)** Comparisons of the frequency of PA defects **(D)**, length of the premaxilla **(E)** and area of the PA **(F)** in chicken embryos from the control, Baicalin, HG, and HG + Baicalin groups. Abbreviations: HG, high glucose; SQ, squamosal bone; PA, parietal bone. Scale bars = 1 mm in **(B)**.

**FIGURE 3:**
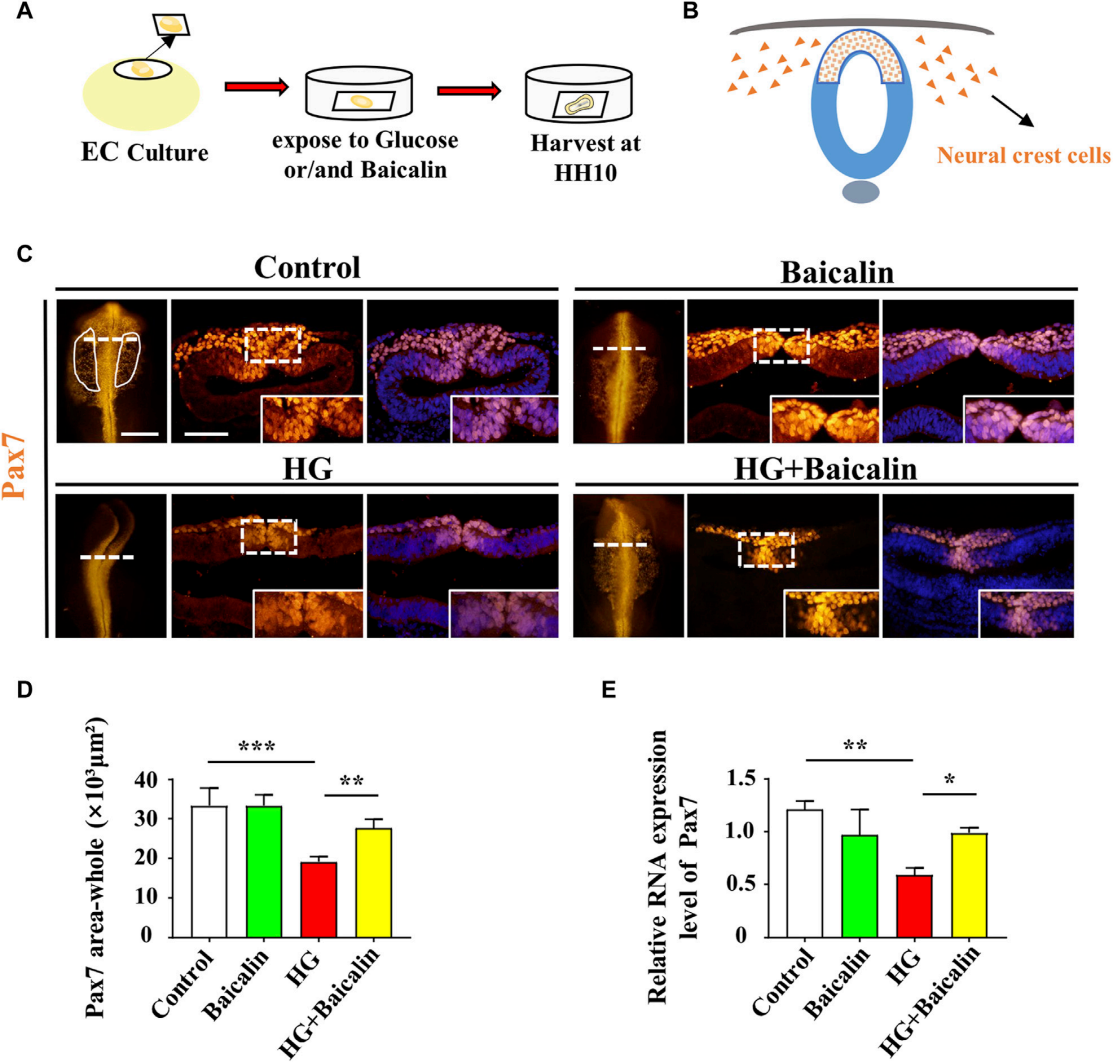
Assessing Pax7-labeled NCCs delaminated from chicken embryos exposed to HG and/or baicalin **(A)** A schematic illustration showing that gastrula chicken embryos were incubated with HG and/or baicalin from HH0 to HH10 in EC culture. **(B)** A schematic of the distribution of early NCCs on the neural tube. **(C)** Representative images of the cranial region of whole-mount Pax7 immunofluorescence, as well as the corresponding cross-sections at the levels indicated by dotted lines from the control, Baicalin, HG, and HG + Baicalin groups. **(D)** Comparisons of Pax7^+^ areas in whole-mount cranial regions of chick embryos from the control, Baicalin, HG, and HG + Baicalin groups. **(E)** Quantitative PCR data showing the mRNA expression of Pax7 in chicken embryos from the control, Baicalin, HG, and HG + Baicalin groups. Abbreviations: HG, high glucose. Scale bars = 200 μm in whole-mount; 100 μm in cross-section of **(C)**.

Fertilized chick eggs were obtained from the Avian Farm of the South China Agriculture University. The eggs were incubated until the chick embryos reached the desired developmental HH stage (Hamburger and Hamilton, 1992) in a humidified incubator (Yiheng Instrument, Shanghai, China) at 38°C and 70% humidity. For the later stage chick embryos, 1.5-day preincubated chick embryos were exposed to either different concentrations of baicalin (Santa Cruz Biotechnology, Dallas, TX, United States), 50 mM glucose (Sigma, United States), or the same amount (approximately 200 µL) of 50 mM mannitol (osmotic control, Sigma, United States) through careful injection into windowed eggs *in vivo* (Figure 2A). For early gastrula embryos, HH0 (Hamburger and Hamilton, 1992) chick embryos were prepared and incubated with 50 mM mannitol, 6 μM baicalin, or/and 50 mM glucose (Wang et al., 2015) using early chick culture (EC culture) (Figure 3A) (Chapman et al., 2001).

### Alizarin red staining of whole embryos

The craniofacial skeleton was visualized in 12-day (E12) chick embryos by staining with alizarin red dye (Solarbio, Beijing, China). Briefly, the embryos were fixed in 95% ethanol for 3 days, after which the skin and viscera were carefully removed before they were postfixed in 95% ethanol for 1 day. The embryos were then treated with 0.5% KOH (Jinan University, Guangzhou, China) for 48 h and stained with 0.001% alizarin red dye for 3 days. Finally, the embryos were cleared in a graded series of glycerol (diluted with water), and the craniofacial skeleton was photographed using a stereomicroscope (Olympus MVX10, Japan).

### Immunofluorescence staining

Chick embryos were harvested after a given incubation time and fixed in 4% PFA overnight at 4°C. Immunofluorescence staining was performed on either whole-mount embryos or transverse sections using the following antibodies: Pax7 (1:200, DSHB, United States), E-cadherin (1:200, DSHB, United States), N-cadherin (1:200, DSHB, United States), and Cadherin6B (1:100, DSHB, United States), and HNK-1 (1:400, Sigma, United States). Briefly, the fixed embryos or transverse sections were then incubated with these primary antibodies at 4°C overnight on a shaker. Following extensive washing, the embryos were incubated with an anti-rabbit IgG conjugated to Alexa Fluor 488 or Alexa Fluor 555 overnight at 4°C on a rocker. All the samples were later counterstained with DAPI (1:1000, Invitrogen, United States) at room temperature for 1 h.

### *In situ* hybridization

Whole-mount *in situ* hybridization of chick embryos was performed according to a standard *in situ* hybridization protocol (Henrique et al., 1995). Digoxigenin-labeled probes were synthesized against Slug, Msx1, FoxD3, and FGF8 (Li et al., 2022). The stained whole-mount chick embryos were photographed by a stereomicroscope (Olympus MVX10, Tokyo, Japan) and then prepared for cryosectioning at a thickness of 16 μm on a cryostat microtome (Leica CM 1900).

### RNA isolation and quantitative PCR

Total RNA was extracted from chick embryos using a TRIzol kit (Invitrogen, United States). PCR amplification of the cDNA was implemented using the corresponding specific primers (note: the sequences are provided in Supplementary Table S4). The PCRs were implemented in a Bio-Rad S1000TM Thermal cycler (Bio-Rad, United States).

### Drug-target interaction prediction

For target prediction, the SMILES of baicalin from PubChem (https://pubchem.ncbi.nlm.nih.gov/) was employed to visualize the structure and predict the target on the Swiss Target Prediction website (http://www.swisstargetprediction.ch/).

### Protein-protein interaction (PPI) network analysis

STRING (Search Tool for the Retrieval of Interacting Genes/Proteins, https://string-db.org) was used for protein-protein interaction (PPI) network analysis.

### Molecular docking

The crystal structure of AKR1B1 (PDB: 1AZ1) was downloaded from the RSCB protein data bank (PDB) (http://www.rcsb.org/). Before molecular docking, open-source PyMOL (https://pymol.org) was employed to remove nonstandard amino acids, crystal water, and impurity chains from the initial structure of AKR1B1 and then hydrogenate and recalculate the charge. AutoDock Tools (ADT, version 4.2.6) was used to convert the protein into the PDBQT format. The structure of Baicalin (Molecule ID: MOL002776) was obtained from TCMSP and converted into mol2 format. Then, molecular docking studies were performed using AutoDock-Vina 1.1.2-. PyMOL 2.3.0 and Discovery Studio 2016 Client were used to visualize the best conformations and analyze the molecular interactions.

### Cell culture and cellular thermal shift assay (CETSA)

The SH-Y5Y cell line (human neuroblastoma cells) was cultured in high glucose DMEM (Gibco, United States, 4.5 g/mL glucose), which contained 10% FBS (fetal bovine serum, ExCell, Shanghai, China) and 1% P/S (double antibody: penicillin 100 U/mL + streptomycin 100 μg/mL, Gibco, United States). Then, the flask was placed in an incubator at 37°C and 5% CO2. The cells were cultured *in vitro* and divided into two groups (control and baicalin). After baicalin treatment, the cells were lysed, supernatant was removed after centrifugation for gradient heating, and the proteins were denatured and precipitated by heating and removed by centrifugation. Western blotting was used to detect and quantify AKR1B1, and expression curves were drawn.

### Western blot

The proteins were isolated from the cells via homogenates using a radio-immuno-precipitation assay (RIPA, Sigma, MO, United States). Western blotting was implemented based on the standard procedure as previously described (Wang et al., 2018b). Primary antibodies were used to detect the expression of AKR1B1 (1:1500, Proteintech, 15439-1-AP, Wuhan Sanying). The loading control was a β-actin antibody (1:1000, Abcam, ab32572, New Territories, HK). After incubation with secondary antibodies (HRP-conjugated goat anti-rabbit IgG 1:4000, EarthOx, 7074S, Millbrae, United States), the samples were developed with SuperSignalTM West Femto Chemiluminescent Substrate (ThermoFisher, Rockford, United States) and the Gel Doc™ XR + System (Bio-Rad, CA, United States).

### Data analysis

The various markers’ positive cell rates and relative mRNA expressions in the different experimental samples were analyzed using Image Pro-Plus 5.0 software (He et al., 2014). Statistical analysis was performed using the SPSS 13.0 statistical package program for Windows. The data are presented as the mean ± SE. Statistical significance was assessed by one-way ANOVA and Tukey’s multiple comparisons test. *p* < 0.05 was considered to be statistically significant.

## Results

### Meta-analysis revealed the correlation between hyperglycemia in pregnancy and craniofacial malformation

First, to systemically assess the correlation between hyperglycemia in pregnancy and craniofacial malformation, we employed a meta-analysis (Gurevitch et al., 2018) at the four typical steps (identification, screening, eligibility, and inclusion) on the literature available in PubMed, Embase, Cochrane Library and Web of Science databases (Figure 1A). In the final included studies (Janssen et al., 1996; Moore et al., 2000; Åberg et al., 2001; Stott-Miller et al., 2010; Balsells et al., 2012; Trindade-Suedam et al., 2016; Yang et al., 2019; Tinker et al., 2020; Maniglio et al., 2022; Zhang et al., 2022), we selected all types of deformities involved in the development of head and facial bones, such as orofacial clefts, cleft lip and/or palate, and positional plagiocephaly. In the context of type I or II or gestational diabetes (Figure 1B). In the context of hyperglycemia in pregnancy, the effect size (ES) at the risk of fetal craniofacial abnormalities was 1.98 [95% confidence interval (CI): 1.39, 2.83] (Figure 1B). Publication bias was evaluated using funnel plots and Egger’s regression test, and the results are shown in Supplementary Figure S1. Taken together, the results of the meta-analysis demonstrated that there are almost certainly associations between diabetes in pregnancy and craniofacial abnormalities, as shown here, but it is simultaneously suggested that there is still a lack of an effective therapeutic approach, which is at least partially due to the underlying biological mechanisms having not yet been completely elucidated. Therefore, we summarized and classified various potential mechanisms of craniofacial abnormalities caused by hyperglycemia during pregnancy mentioned in the 10 included studies, including impaired oxidative stress and glucose tolerance, as shown in Figure 1C.

### Baicalin demonstrated its capacity to reverse the craniofacial malformation induced by high glucose

Our previous study not only revealed the antioxidant effects of baicalin but also confirmed its protective effect on embryonic development in a hyperglycemic environment (Wang et al., 2018a; Wang et al., 2020). To assess the impact of baicalin on countering craniofacial malformation induced by high glucose during pregnancy, we exposed chicken embryos to HG or HG + baicalin before harvesting them at 12 days of incubation (Figure 2A). Alizarin red staining was performed on chicken embryos treated with HG and/or baicalin, and then the skulls from the control, baicalin, HG, and HG + baicalin groups were photographed (Figure 2B). The results showed that HG increased the mortality of chick embryos and frequency of parietal bone (PA) defects, and additional application of baicalin reversed this tendency (Figures 2C, D). The corresponding quantitative analysis showed that HG treatment significantly reduced the length of the premaxilla and area of the parietal bone (PA), but these effects were reversed by baicalin treatment (Figures 2E, F). This suggested that baicalin administration was indeed able to efficiently rescue the craniofacial malformation induced by high glucose concentrations during pregnancy.

### Baicalin administration reversed HG-inhibited delamination of cranial NCCs on the dorsal side of the developing neural tube

To examine whether the aforementioned craniofacial malformation phenotype occurred in the early stage of neural crest development, we exposed gastrula chicken embryos to HG (50 mM) and/or baicalin (6 μM) in EC culture and then evaluated neural crest delamination after 42 h of incubation (Figure 3A). Whole-mount Pax7 immunofluorescence staining showed that baicalin did not only affect Pax7-labeled NCC delamination compared to the control, but it could significantly reverse the HG-induced reduction in Pax-7-labeled NCC delamination (Figures 3C, D), which was verified by quantitative PCR data (Figure 3E).

**FIGURE 4:**
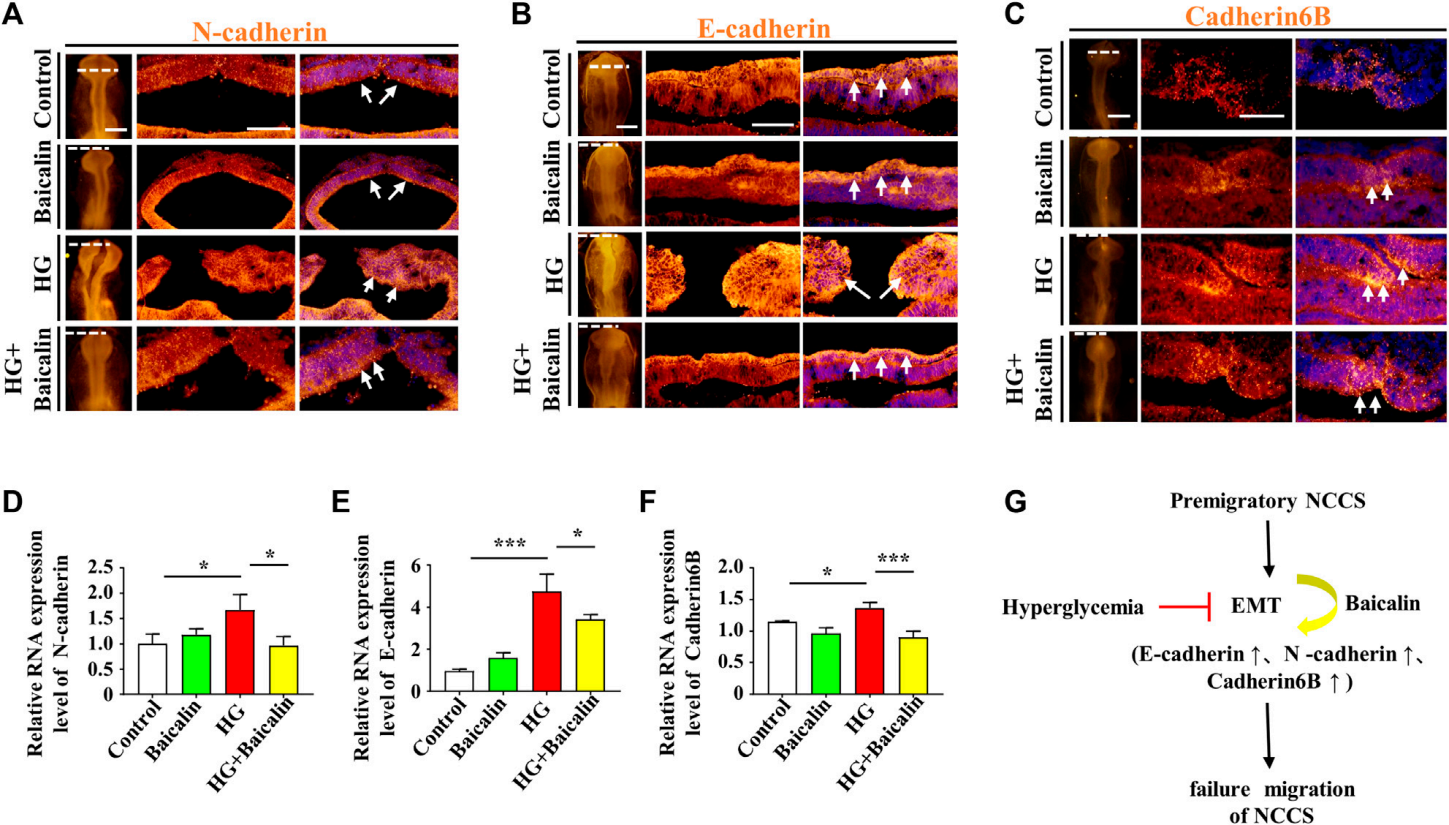
Assessing the expression of E-cadherin, N-cadherin, and cadherin 6B in the cranial regions of chicken embryos exposed to HG and/or baicalin **(A–C)** Representative images of the cranial region of whole-mount immunofluorescence of N-cadherin **(A)**, E-cadherin **(B)** and cadherin 6B **(C)**, as well as the corresponding cross-sections at the levels indicated by dotted lines from the control, Baicalin, HG, and HG + Baicalin groups. **(D–F)** Quantitative PCR data showing the mRNA expression of N-cadherin **(D)**, E-cadherin **(E)** and cadherin 6B **(F)** in chicken embryos from the control, Baicalin, HG, and HG + Baicalin groups. **(G)** The diagram indicates that baicalin rescues the early EMT process inhibited by a HG environment. Abbreviations: HG, high glucose. Scale bars = 200 μm in whole mounts of **(A–C)**; 100 μm in cross-sections of **(A–C)**.

**FIGURE 5:**
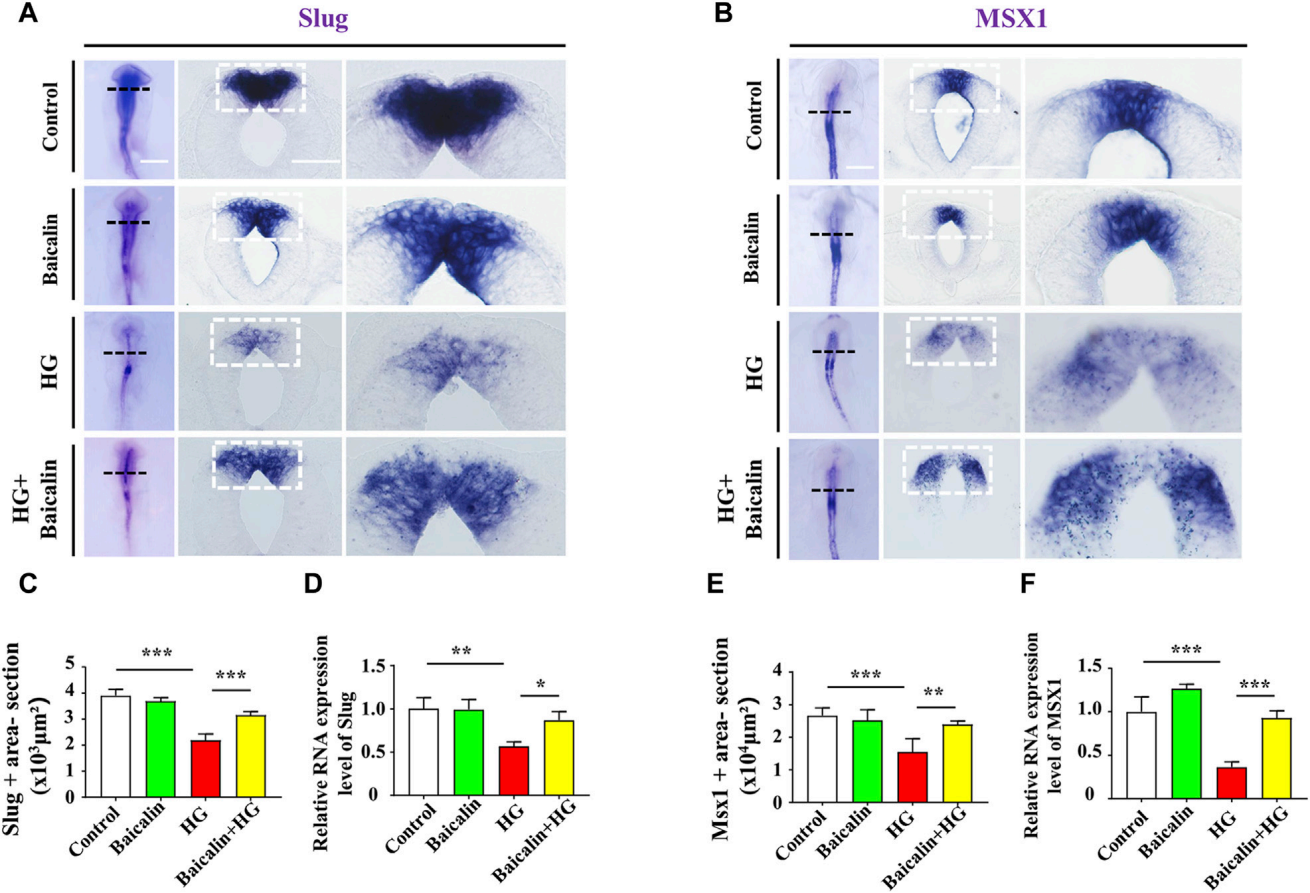
Assessing the expression of Slug and Msx1 in the cranial regions of chicken embryos exposed to HG and/or baicalin **(A)** Representative images of the cranial region of whole-mount Slug *in situ* hybridization, as well as the corresponding cross-sections at the levels indicated by dotted squares from the control, Baicalin, HG, and HG + Baicalin groups. **(B)** Representative images of the cranial region of whole-mount Msx1 *in situ* hybridization, as well as the corresponding cross-sections at the levels indicated by dotted squares from the control, Baicalin, HG, and HG + Baicalin groups. **(C)** Bar chart showing the comparisons of Slug^+^ areas in the cranial regions of chicken embryos from the control, Baicalin, HG, and HG + Baicalin groups. **(D)** Quantitative PCR data showing the mRNA expression of Slug in chicken embryos from the control, Baicalin, HG, and HG + Baicalin groups. **(E)** Bar chart showing the comparisons of Msx1^+^ areas in the cranial regions of chicken embryos from the control, Baicalin, HG, and HG + Baicalin groups. **(F)** Quantitative PCR data showing the mRNA expression of Msx1 in chicken embryos from the control, Baicalin, HG, and HG + Baicalin groups. Abbreviations: HG, high glucose. Scale bars = 200 μm in whole mounts of **(A,B)**; 100 μm in cross-sections of **(A,B)**.

Loss of expression of EMT-related adhesion molecules is indispensable for EMT in NCC delamination (Gheldof and Berx, 2013). Whole-mount immunofluorescence staining against adhesion molecules, including N-cadherin, E-cadherin, and Cadherin 6B, was subsequently carried out (Figures 4A–C), and the results showed that the addition of baicalin could reverse the HG-increased expression of N-cadherin, which was confirmed by quantitative PCR data (Figure 4D), as well as E-cadherin and Cadherin 6B (Figures 4E, F). To examine whether the expression of EMT-related transcription factors (Duband et al., 1995) was affected, we implemented whole-mount *in situ* hybridization against Slug and Mxs1 in embryos exposed to HG or/and baicalin (Figures 5A, B). Baicalin significantly reversed the inhibition of HG-inhibited Slug (Figure 5C) and Msx1 (Figure 5E) expression on the dorsal side of neural tubes. The quantitative PCR data were generally the same as those from *in situ* hybridization (Figures 5D, F). This implied that baicalin could successfully rescue HG-inhibited NCC delamination by regulating the expression of EMT-related transcription factors and adhesion molecules.

### Baicalin administration reversed HG-inhibited migration of cranial NCCs in cranial neural crest development

**FIGURE 6:**
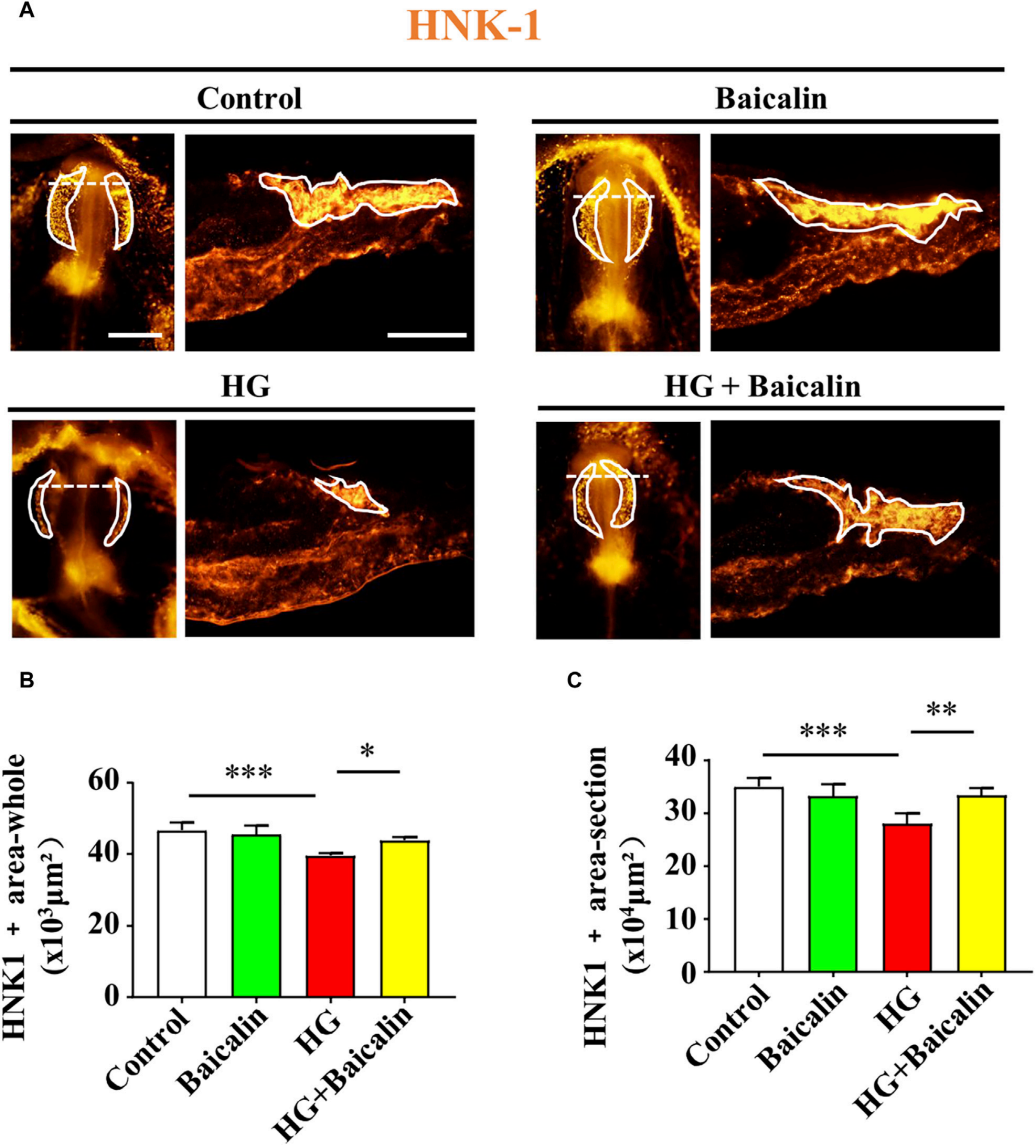
Assessing HNK-1-labeled migratory NCCs from chicken embryos exposed to HG and/or baicalin **(A)** Representative images of the cranial region of whole-mount HNK-1 immunofluorescence, as well as the corresponding cross-sections at the levels indicated by dotted lines from the control, Baicalin, HG, and HG + Baicalin groups. **(B,C)** Bar chart showing the comparisons of HNK-1^+^ areas in whole-mount **(B)** and histological sections **(C)** of chicken embryos from the control, Baicalin, HG, and HG + Baicalin groups. Scale bars = 200 μm in whole mount of **(A)**; 100 μm in cross-section of **(A)**.

To determine whether the migration of NCCs in cranial neural crest development was affected, we implemented whole-mount immunofluorescence staining against HNK-1 (Bronner-Fraser, 1986) on chicken embryos treated with HG and/or baicalin. The results clearly indicated that combinational application of baicalin could resume HG-inhibited HNK-1-labeled NCC migration at both the whole-mount and section levels (Figures 6A–C).

**FIGURE 7:**
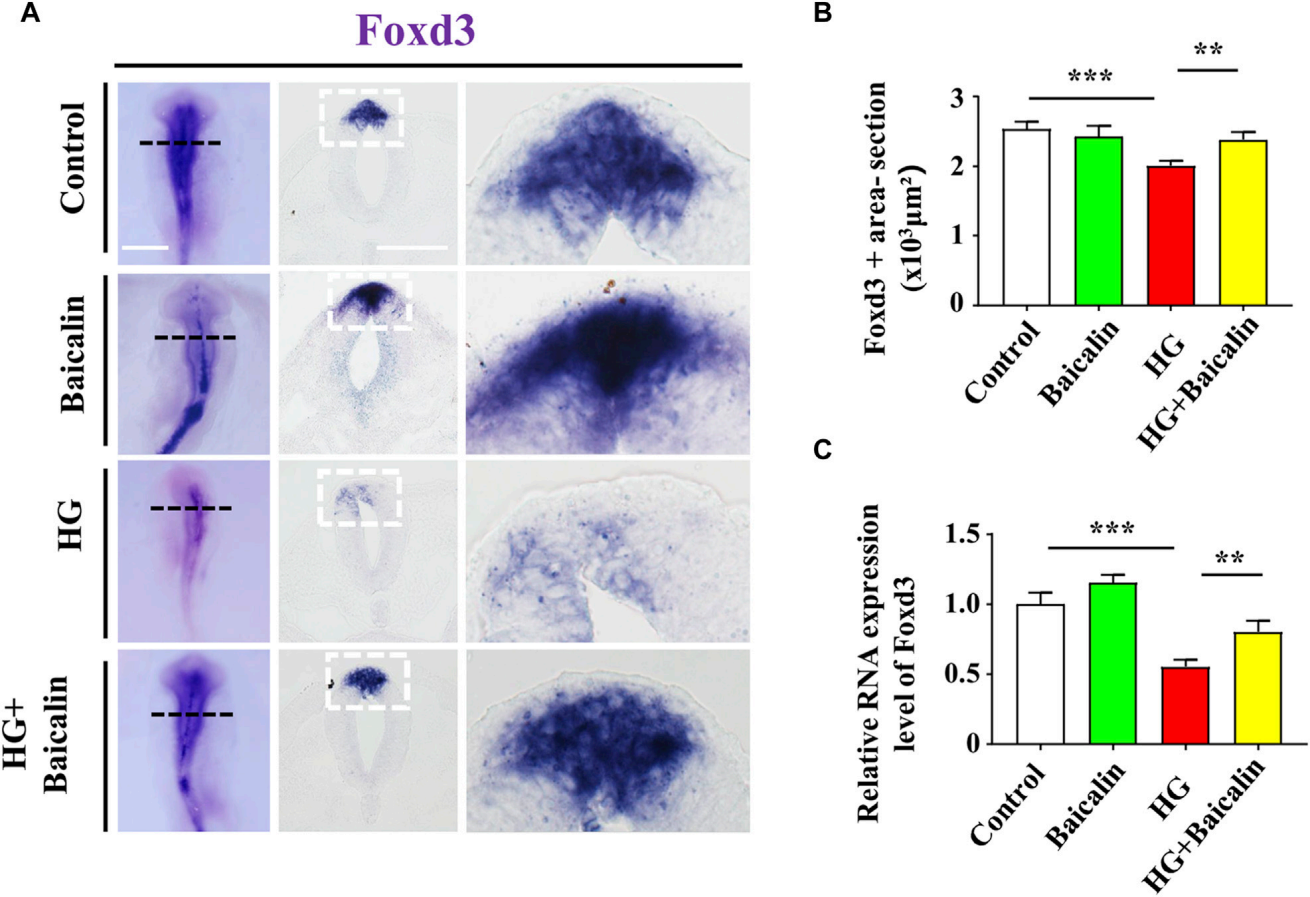
Assessing the expression of Foxd3 in the cranial regions of chicken embryos exposed to HG and/or baicalin **(A)** Representative images of the cranial region of whole-mount Foxd3 *in situ* hybridization, as well as the corresponding cross-sections at the levels indicated by dotted squares from the control, Baicalin, HG, and HG + Baicalin groups. **(B)** Bar chart showing the comparisons of Foxd3^+^ areas in the cranial regions of chicken embryos from the control, Baicalin, HG, and HG + Baicalin groups. **(C)** Quantitative PCR data showing the mRNA expression of Foxd3 in chicken embryos from the control, Baicalin, HG, and HG + Baicalin groups. Scale bars = 200 μm in whole mount of **(A)**; 50 μm in cross-sections of **(A)**.

Foxd3 and FGF8 are required for NCC migration (Stewart et al., 2006; Sato et al., 2011), so we carried out Foxd3 immunofluorescence staining after whole-mount Foxd3 *in situ* hybridization (Figure 7A; Supplementary Figure S2A). The combinational application of baicalin significantly reversed the HG-inhibited Foxd3 expression on the dorsal side of the neural tubes (Figure 7B), which was verified by subsequent quantitative PCR data (Figure 7C). However, in contrast to Foxd3 expression, the additional application of baicalin did not rescue HG-inhibited FGF8 expression (Supplementary Figure S2B). This suggests that baicalin administration could rescue HG-inhibited NCC migration through the involvement of Foxd3 gene expression regulation.

### Baicalin may modulate NCC development by specifically binding to AKR1B1

**FIGURE 8:**
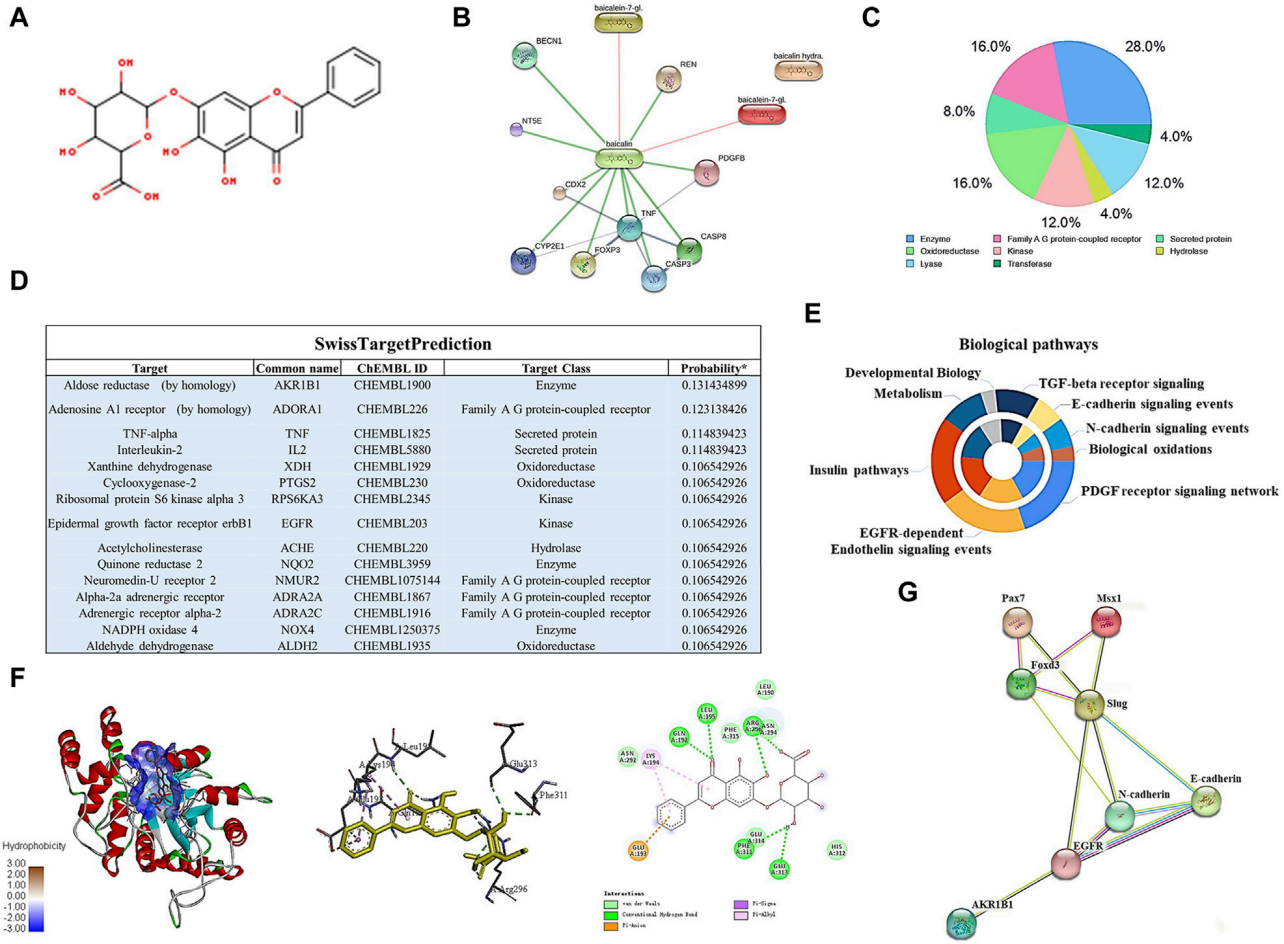
Bioinformatics analysis and target gene prediction of baicalin. **(A)** The two-dimensional structure of baicalin according to SwissTargetPrediction. **(B)** Prediction of the potential target protein of baicalin by using the Stitch website. **(C)** Pie chart shows the categories of baicalin-regulated targets based on SwissTargetPrediction. **(D)** The table shows the information about the predicted most likely targets for baicalin. **(E)** The enrichment and anlysis of potential target genes and proteins of baicalin from **(B,D)** for different biological pathways. **(F)** The proposed three- and two-dimensional binding models of the interaction between baicalin and AKR1B1 derived from Open-Source PyMOL and Discovery Studio 2016 Client software. In the three-dimensional interaction, baicalin is presented by carton, AKR1B1 is presented by ball-stick, and the binding residues are presented by sticks. **(G)** PPI analysis of AKR1B1 and NCC-related genes in this study using the Stitch website.

**FIGURE 9:**
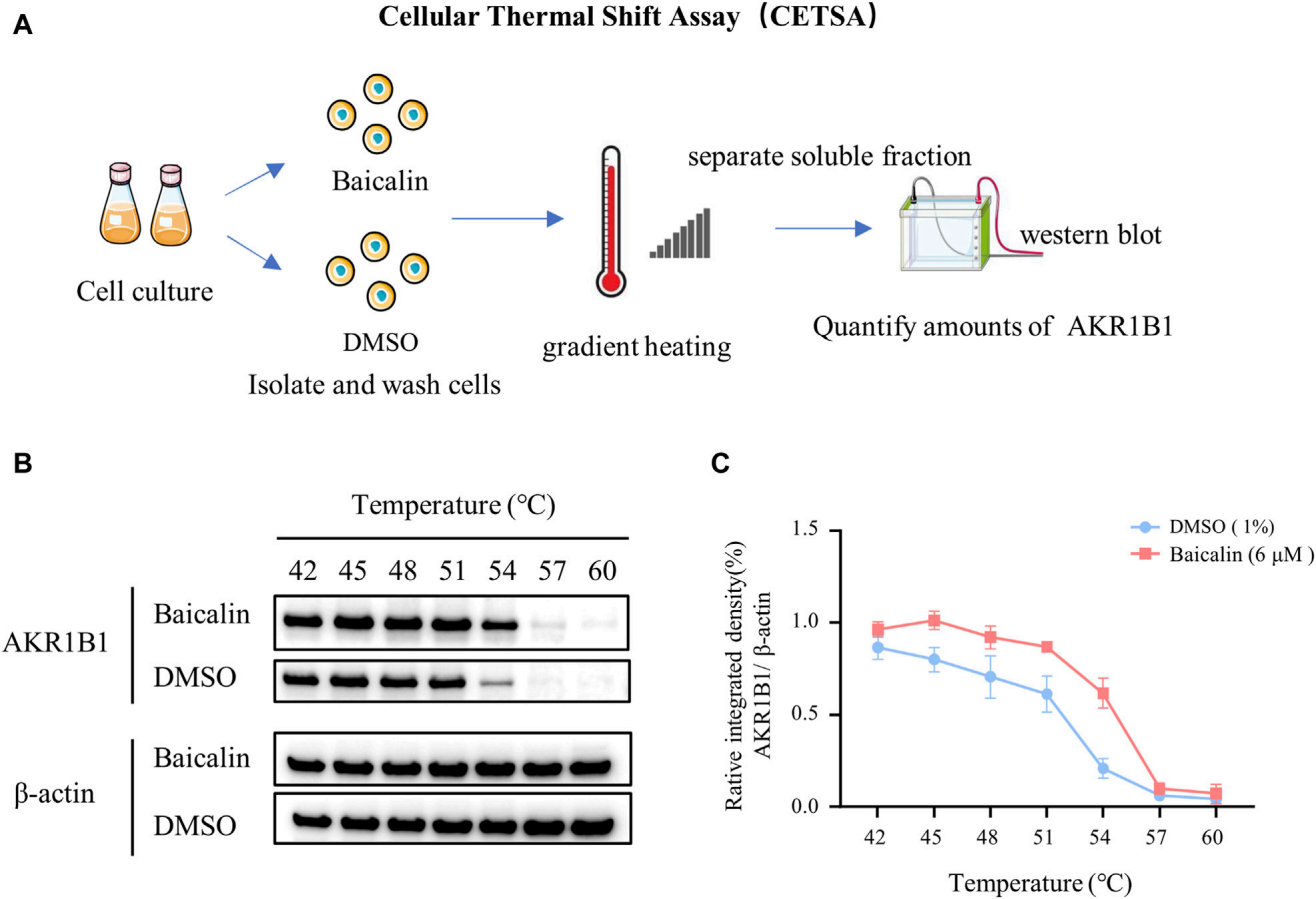
*In vitro* validation—cellular thermal shift assay. **(A)** A schematic illustration showing the process of cell culture *in vitro* and the cellular thermal shift assay. **(B)** Western blot data showing AKR1B1 expressions at the protein level in cells treated with baicalin or DMSO (control). **(C)** The curves quantified the changes in the expression of AKR1B1 with the increase of temperature.

To identify the potential targets of baicalin, we drew the chemical structure of baicalin (Figure 8A) and predicted its target proteins based on the Stitch website and Swiss Target Prediction (Figures 8B–D), which indicated that 28% of the top 15 targets were associated with the enzyme class. The potential target genes and proteins were enriched and analyzed for different biological pathways (Figure 8E), in which EGFR, PDGF, insulin pathways, TGF-beta, biological oxidations and cell adherins were involved. The most strongly correlated gene was AKR1B1 (Figure 8D). We performed a molecular docking analysis of baicalin and AKR1B1, and the results showed that the binding energy for baicalin and AKR1B1 was −8.4 kcal/mol. The three-dimensional diagram showed that baicalin bound to amino acid residues in the AKR1B1 pocket, where baicalin formed hydrogen bonds with Gln47, pi-sulfur with Tyr45 and an attractive charge with Asp102 (Figure 8F). To confirm the potential link between AKR1B1 and NCC development, bioinformatics analysis was employed to construct a protein‒protein interaction network. The results indicated that AKR1B1 may affect NCC-related genes through EGFR signaling. (Figure 8G). Finally, the direct binding of baicalin to AKR1B1 was further confirmed by an *in vitro* cellular thermal shift assay. With increasing temperature, the expression level of AKR1B1 in cells treated with baicalin was greater than that in the control (DMSO), especially at 54°C. AKR1B1 was verified to be the target of baicalin and to play a protective role intracellularly (Figures 9A–C).

## Discussion

Growing evidence has shown that overt diabetes mellitus during pregnancy is closely associated with a dramatically increased risk of various perinatal disorders in nervous, cardiovascular and musculoskeletal system defects (Moore et al., 2000). In this study, we focused on craniofacial malformation in the presence of hyperglycemia in pregnancy, so a meta-analysis was first employed to comprehensively and systemically review the results of all previous studies in this area. From the view of the meta-analysis results, we could see that both preexisting diabetes before pregnancy and gestational diabetes could increase the risk of various craniofacial malformations but that this risk was worse in patients with preexisting diabetes than in those with gestational diabetes (Figure 1B). This definitely tamped down the previous observation that the exposure to hyperglycemia in pregnancy would adversely impact craniofacial skeleton development, thereby raising the possibility of having congenital and postnatal diseases (Mussatto et al., 2015). Meanwhile, there is no doubt that this highlights an obvious need to seek new medications to prevent or confront the developmental defects induced by diabetes in pregnancy.

As we know, chickens have not gained widespread use as an animal model in diabetes research primarily due to their nonmammalian nature, with their genetic makeup being approximately 70% homologous to that of humans (King, 2012; Shi et al., 2014). Among vertebrates, chicken embryos are favored models in developmental biology. They are easily accessible, have a short incubation period, and exhibit phenotypes similar to mammalian developmental abnormalities in a hyperglycemic environment (Datar and Bhonde, 2005; Datar and Bhonde, 2011; Bozkurt et al., 2021). Therefore, we chose chicken embryos as the model to proceed for our study.

From the literature included in the meta-analysis, we found that there are various mechanisms of craniofacial malformation caused by hyperglycemia, such as oxidative stress and ROS (Figure 1C). Baicalin, an extract of traditional Chinese medicine, has been extensively used as a traditional medicine to reduce inflammation in many East Asian countries for a long time (Ishimaru et al., 1995). In addition to anti-inflammation, baicalin also exhibits many other pharmacological properties, such as anticancer and anti-pruritic effects (Li-Weber, 2009; Trinh et al., 2010), which inspired us to employ it in this study. Another important factor in its favor is that baicalin could go through the placental barrier at different gestational stages after being absorbed in the maternal intestine and entering maternal circulation (Song et al., 2010). Most of the abovementioned effects of baicalin result from its antioxidative and anti-inflammatory effects through upregulating antioxidant enzymes and suppressing NF-κB signaling pathways (Wu et al., 2018). Thus, baicalin is ideal for use in confronting diabetes in pregnancy since diabetes mellitus is regarded as an inflammatory disorder (Tsalamandris et al., 2019). Regarding the potential embryotoxicity of baicalin, our previous study showed that 6 μM baicalin itself did not have a significantly negative influence on embryo development (Wang et al., 2020). After deliberate choice (i.e., baicalin as the chosen compound) and exclusion of embryotoxicity, we first evaluated the effect of 6 μM baicalin on developing chicken embryos by observing the effect of the combined application of HG and baicalin on craniofacial skeleton development, and this *in vivo* experiment clearly showed that baicalin administration could successfully rescue high glucose-induced craniofacial skeleton malformation (Figure 2).

Embryos at an early stage of development are extremely vulnerable to environmentally harmful influences since their own immune system has not yet been well established (Hamdoun and Epel, 2007). Craniofacial bone is principally derived from the cranial neural crest at the early stage of embryo development, so we naturally started our study based on the spatiotemporal development of the neural crest at the initial stage, i.e., NCC delamination, EMT, migration, and differentiation, using a gastrula chicken embryo model. To determine the delamination of NCCs, we employed Pax7 as a marker for premigratory NCC generation (Basch et al., 2006) in gastrula chicken embryos exposed to HG or/and baicalin, mannitol was used as an osmotic control due to the HG. The results unmistakably demonstrated that baicalin administration indeed significantly rescued high glucose-restricted NCC production (Figure 3). In subsequent experiments to decipher the underlying mechanism, we found that baicalin could downregulate the HG-increased expression of adhesion molecules (N-cadherin, E-cadherin and cadherin 6B) in cranial neural tubes (Figure 4), indicating that the high glucose-restricted EMT process and NCC delamination were lifted by baicalin administration. It is widely known that EMT is intensely associated with a reduction in cell‒cell adhesion, i.e., the expression of these adhesion molecules must be downregulated, which is controlled by some EMT-related transcription factors, such as the zinc finger gene Slug (Duband et al., 1995). Likewise, we observed that the HG-suppressed expression of Slug and Msx1 on the dorsal side of neural tubes bounced back after baicalin administration (Figure 5), implying that baicalin administration is conducive to restoring the expression of these key EMT-related transcription factors, thereby promoting the process of neural crest EMT and delamination. After NCC delamination, NCCs migrate long distances to populate various peripheral sites. Immunofluorescence staining of HNK-1 was utilized to identify the influence of migratory NCCs by baicalin administration, and the results showed that the HG-inhibited HNK-1-labeled NCC migration was significantly rescued by baicalin administration (Figure 6). Therefore, what is the expression of genes controlling NCC migration in this scenario? For this purpose, we detected the expression of Foxd3 and FGF8 (Thomas and Erickson, 2009) in chicken embryos exposed to HG and/or baicalin, and the results showed that HG-inhibited Foxd3 expression increased again following baicalin administration (Figure 7), suggesting gene expression evidence underlying NCC migration improvement.

Finally, to further explore the mechanism of baicalin, we used the molecular informatics website to predict the potential target genes and proteins of baicalin and carried out enrichment analysis of these genes in biological pathways. The gene with the highest correlation was AKR1B1, which has been associated with multiple complications of diabetes and plays a role in ROS production and promoting EMT (Wu et al., 2017; Wu et al., 2020). We used molecular docking software to simulate docking with baicalin molecules and obtained ideal results. Molecular docking simulations also showed ideal results for the interaction between baicalin and AKR1B1. Therefore, we hypothesize that baicalin may target AKR1B1 to rescue craniofacial malformations in hyperglycemic pregnancy. However, AKR1B1 may influence the key genes involved in the epithelial-mesenchymal transition (EMT) process of neural crest cells through EGFR signaling (Zhang et al., 2021), a crucial pathway in NCC development (Gassmann et al., 1995; Golding et al., 2000). Additionally, Wu et al. (2020) suggested that the overexpression of AKR1B1 could suppress AMPK activation, thus promoting the EMT process in lens epithelial cells during diabetic cataract formation.

In recent years, the cell heat transfer assay (CETSA) has become widely used in drug discovery workflows. CETSA based on Western blot is mainly used to verify the target binding of a molecule to its target protein (Tolvanen, 2022). Therefore, we performed a CETSA of baicalin and AKR1B1 in our final research. Surprisingly, the thermal stability of AKR1B1 in cells treated with baicalin was significantly greater than that in the control (DMSO), which was manifested as an increase in AKR1B1 expression, further confirming our speculation that baicalin can play a protective role in cells by targeting on AKR1B1 (Figure 9).

In summary, baicalin was chosen to confront high glucose condition was firstly verified in skull formation by exposing chicken embryos to the combined application of HG and baicalin. Based on the events in NCC development, baicalin administration was found to reverse HG-inhibited NCC delamination and EMT, as well as the expression of corresponding adhesion molecules and key EMT-related transcription factors. Additionally, HG-restricted NCC migration was significantly rescued by baicalin administration. Molecular docking and cell heat transfer assay revealed the possible target gene AKR1B1 of baicalin in confronting the craniofacial skeleton malformation caused by high glucose. The study suggests that baicalin’s significant effect on confronting hyperglycemia-induced craniofacial skeleton malformation occurs mainly at the early stage of NCC development, although more comprehensive studies are needed to reveal the precise mechanism.

## Data Availability

The datasets presented in this study can be found in online repositories. The names of the repository/repositories and accession number(s) can be found in the article/Supplementary Material.
