# Supplementary material for: Baicalin administration could rescue high glucose-induced craniofacial skeleton malformation by regulating neural crest development
Source: Front Pharmacol. 2024 Mar 7;15:1295356. doi: 10.3389/fphar.2024.1295356 (PMC10955141; doi:10.3389/fphar.2024.1295356)
Supplement: Supplementary file 1 [file DataSheet1.docx]

**Supplementary Fig. 1.** Publication bias of the meta-analysis was evaluated using funnel plots (A) and Egger’s regression test (B).

**Supplementary Fig. 2. *Assessing the expression of FGF8 in the cranial regions of chicken embryos exposed to HG and/or baicalin***

A: Representative images of the cranial region of whole-mount FGF8 *in situ* hybridization, as well as the corresponding cross-sections at the levels indicated by dotted squares from the control, baicalin, HG, and HG+Baicalin groups. **B:** Quantitative PCR data showing the mRNA expression of FGF8 in chicken embryos from the control, baicalin, HG, and HG+Baicalin groups. Scale bars = 200 μm in whole mount of A; 50 μm in cross-sections of A.

**Supplementary Table 1.** Summary of Included Studies in the meta-analysis

**Supplementary Table 2.** Quality assessment of cross-sectional studies based on guidelines from the NOS statement.

**Supplementary Table 3.** Quality assessment of cohort studies based on guidelines from the NOS statement.

**Supplementary Table 4.** The primer sequences used in this study.


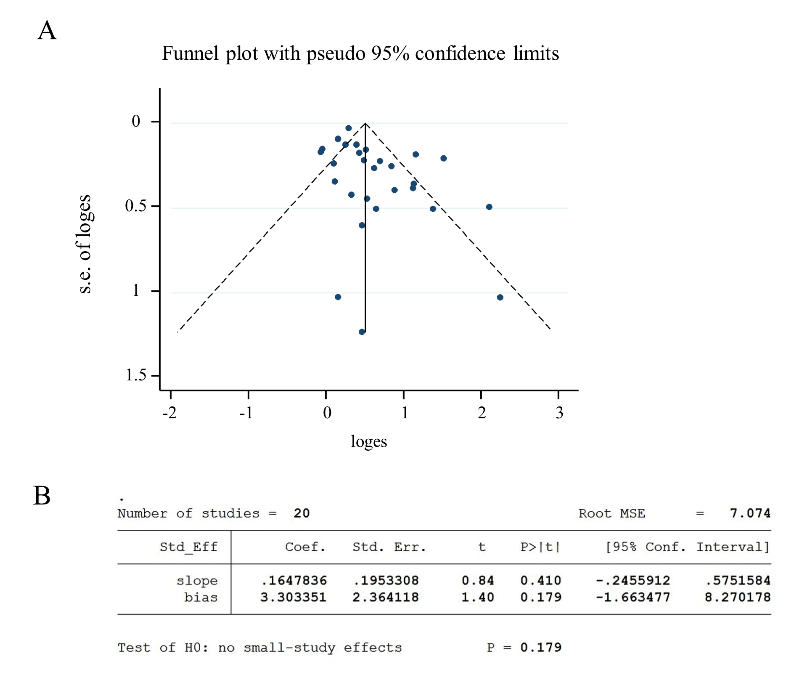


Supplementary Figure 1


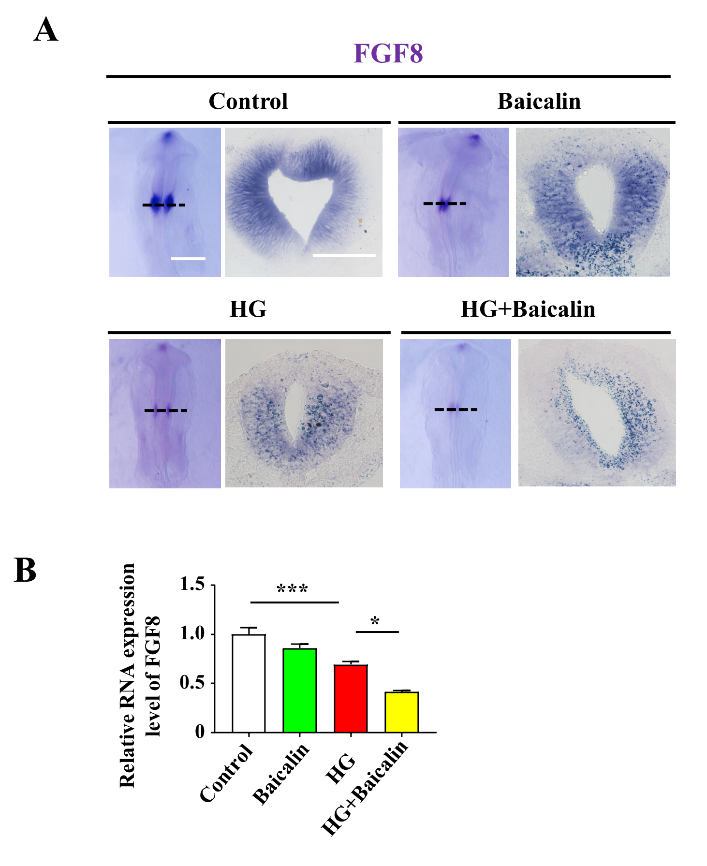


Supplementary Figure 2


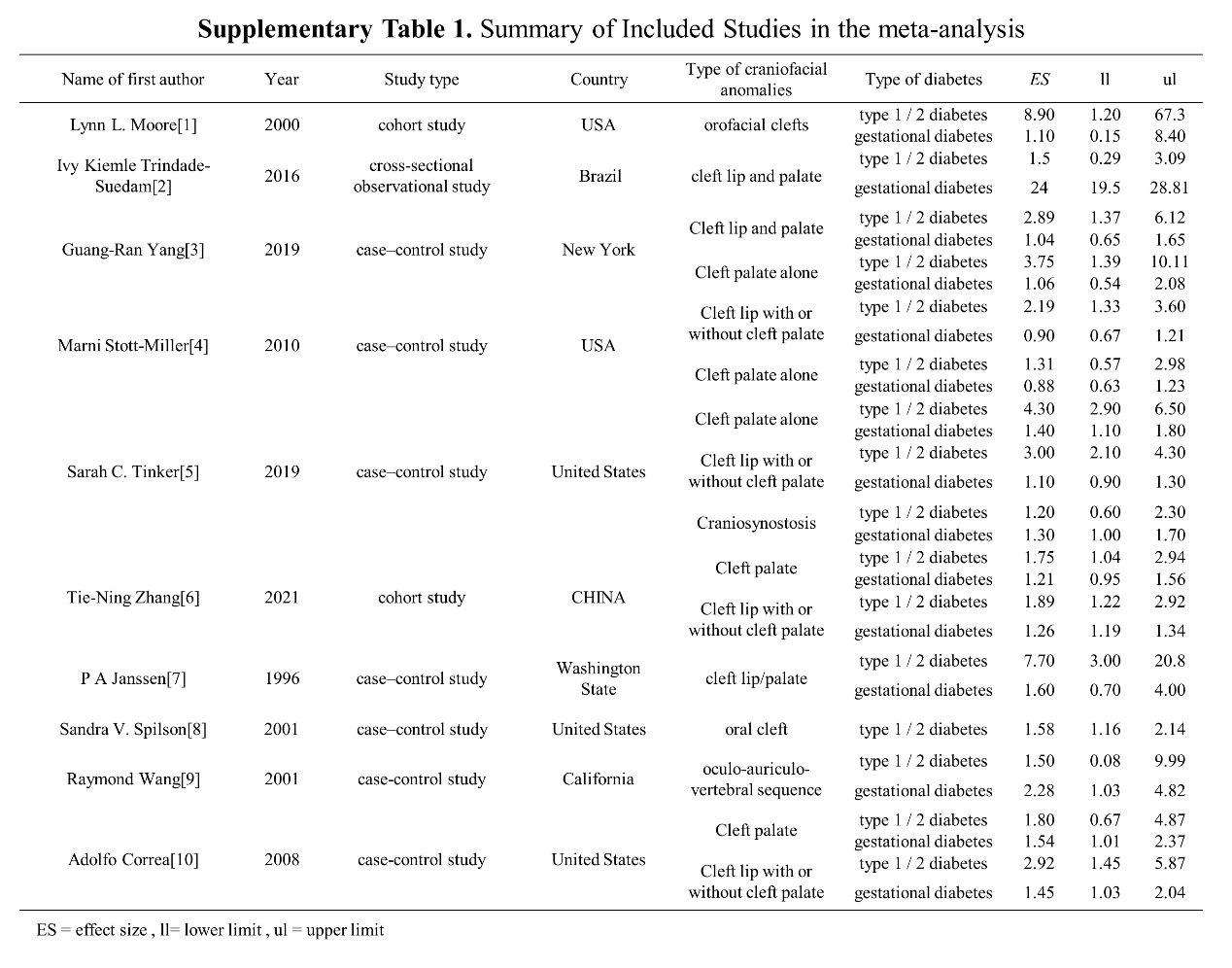


Supplementary Table 1


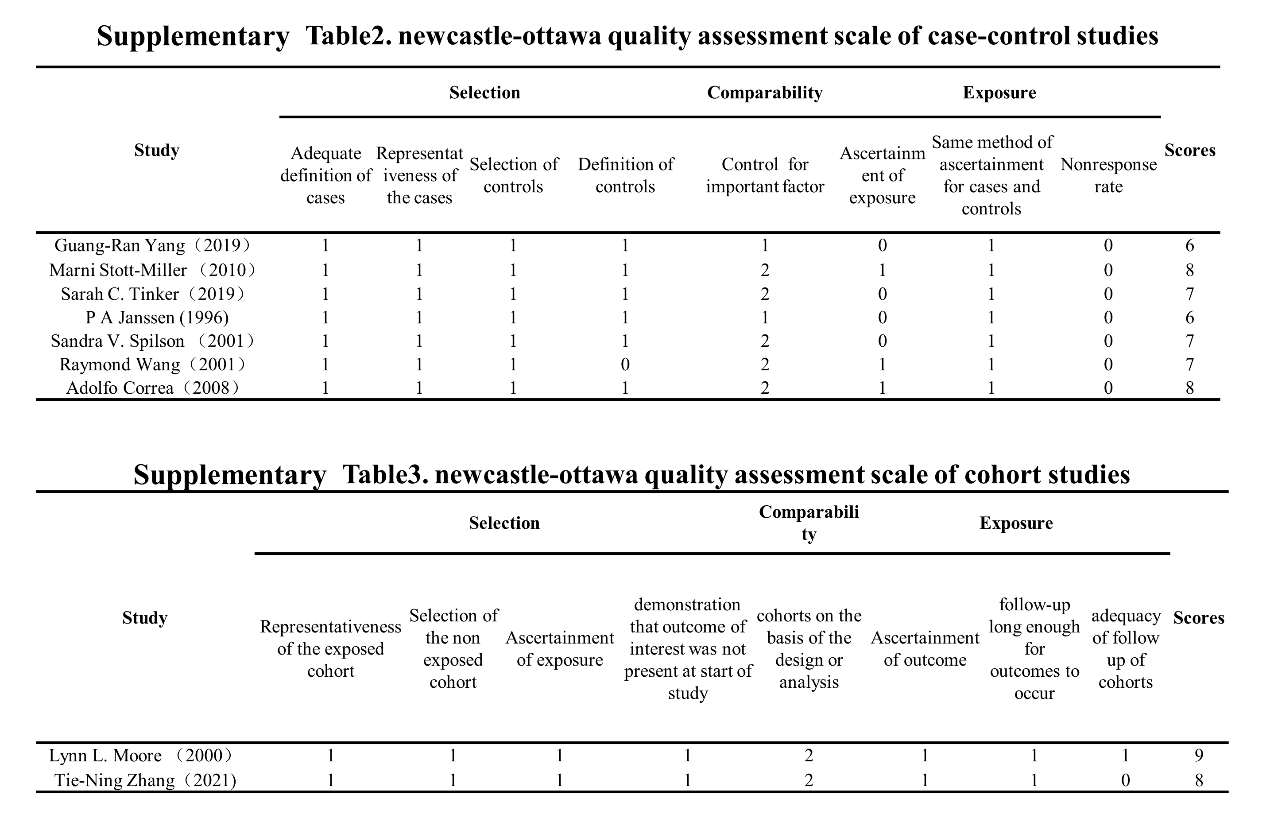


Supplementary Table 2、3


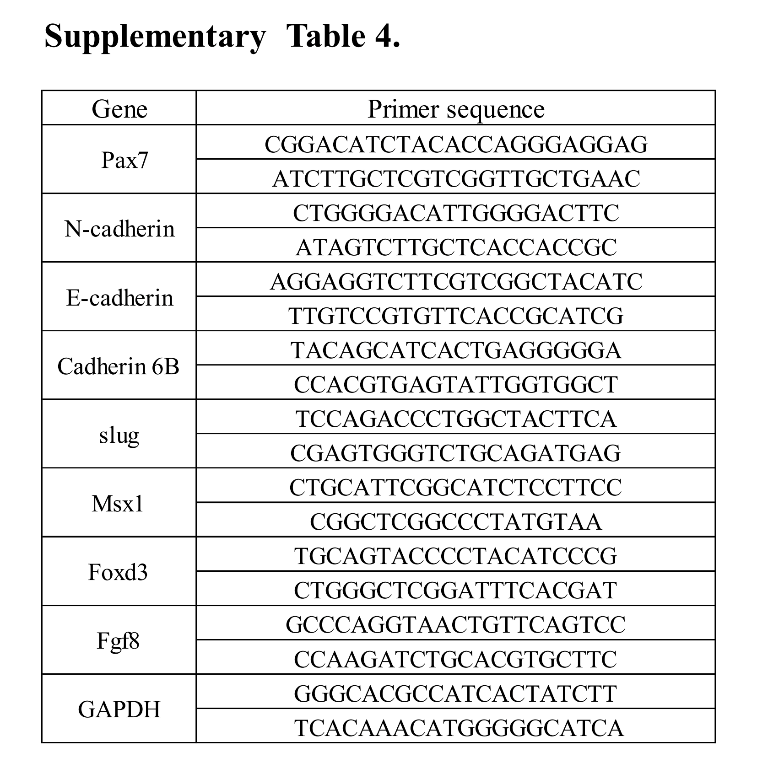


Supplementary Table 4

**Reference**

1. L.L. Moore, M.R. Singer, M.L. Bradlee, K.J. Rothman, and A. Milunsky. A prospective study of the risk of congenital defects associated with maternal obesity and diabetes mellitus. Epidemiology:689-694 (2000).

2. A. Åberg, L. Westbom, and B. Källén. Congenital malformations among infants whose mothers had gestational diabetes or preexisting diabetes. Early human development. 61:85-95 (2001).

3. I.K. Trindade-Suedam, L.M.v. Kostrisch, L.A.F. Pimenta, C.A. Negrato, S.B. Franzolin, and A.S. Trindade Junior. Diabetes mellitus and drug abuse during pregnancy and the risk for orofacial clefts and related abnormalities. Revista latino-americana de enfermagem. 24: (2016).

4. G.-R. Yang, T.D. Dye, and D. Li. Effects of pre-gestational diabetes mellitus and gestational diabetes mellitus on macrosomia and birth defects in Upstate New York. Diabetes Research and Clinical Practice. 155:107811 (2019).

5. M. Balsells, A. García‐Patterson, I. Gich, and R. Corcoy. Major congenital malformations in women with gestational diabetes mellitus: a systematic review and meta‐analysis. Diabetes/metabolism research and reviews. 28:252-257 (2012).

6. M. Stott‐Miller, C.L. Heike, M. Kratz, and J.R. Starr. Increased risk of orofacial clefts associated with maternal obesity: case–control study and Monte Carlo‐based bias analysis. Paediatric and perinatal epidemiology. 24:502-512 (2010).

7. S.C. Tinker, S.M. Gilboa, C.A. Moore, D.K. Waller, R.M. Simeone, S.Y. Kim, D.J. Jamieson, L.D. Botto, J. Reefhuis, and N.B.D.P. Study. Specific birth defects in pregnancies of women with diabetes: National Birth Defects Prevention Study, 1997–2011. American journal of obstetrics and gynecology. 222:176. e171-176. e111 (2020).

8. P. Maniglio, M. Noventa, S. Tartaglia, M. Petracca, M. Bonito, E. Ricciardi, G. Ambrosini, G. Buzzaccarini, and A.S. Laganà. The Obstetrician Gynecologist’s role in the screening of infants at risk of severe plagiocephaly: Prevalence and risk factors. European Journal of Obstetrics & Gynecology and Reproductive Biology. 272:37-42 (2022).

9. T.-N. Zhang, X.-M. Huang, X.-Y. Zhao, W. Wang, R. Wen, and S.-Y. Gao. Risks of specific congenital anomalies in offspring of women with diabetes: A systematic review and meta-analysis of population-based studies including over 80 million births. PLoS medicine. 19:e1003900 (2022).

10. P.A. Janssen, I. Rothman, and S.M. Schwartz. Congenital malformations in newborns of women with established and gestational diabetes in Washington State, 1984–91. Paediatric and perinatal epidemiology. 10:52-63 (1996).
